# Pre-/analytical factors affecting whole blood and plasma glucose concentrations in loggerhead sea turtles (*Caretta caretta*)

**DOI:** 10.1371/journal.pone.0229800

**Published:** 2020-03-03

**Authors:** Patricia E. Kunze, Justin R. Perrault, Yu-Mei Chang, Charles A. Manire, Samantha Clark, Nicole I. Stacy

**Affiliations:** 1 Royal Veterinary College, University of London, London, United Kingdom; 2 Institute of Zoology, Zoological Society of London, London, United Kingdom; 3 Loggerhead Marinelife Center, Juno Beach, Florida, United States of America; 4 Department of Comparative, Diagnostic, and Population Medicine, Aquatic, Amphibian, and Reptile Pathology Program, College of Veterinary Medicine, University of Florida, Gainesville, FL, United States of America; University of Minnesota, UNITED STATES

## Abstract

Blood glucose is vital for many physiological pathways and can be quantified by clinical chemistry analyzers and in-house point-of-care (POC) devices. Pre-analytical and analytical factors can influence blood glucose measurements. This project aimed to investigate pre-analytical factors on whole blood and plasma glucose measurements in loggerhead sea turtles (*Caretta caretta*) by evaluating the effects of storage (refrigeration) up to 48h after sampling and of packed cell volume (PCV) on whole blood glucose analysis by POC glucometer (time series n = 13); and by evaluating the effects of storage (room temperature and refrigeration) on plasma glucose concentrations using a dry slide chemistry analyzer (DCA) at various conditions: immediate processing and delayed plasma separation from erythrocytes at 24h and 48h (time series n = 14). The POC glucometer had overall strong agreement with the DCA (CCC = 0.76, r = 0.84, C_b_ = 0.90), but consistently overestimated glucose concentrations (mean difference: +0.4 mmol/L). The POC glucometer results decreased significantly over time, resulting in a substantial decline within the first 2h (0.41±0.47 mmol/L; 8±9%) that could potentially alter clinical decisions, thereby highlighting the need for immediate analysis using this method. The effects of PCV on glucose could not be assessed, as the statistical significance was associated with one outlier. Storage method significantly affected plasma glucose measurements using DCA, with room temperature samples resulting in rapid decreases of 3.57±0.89 mmol/L (77±9%) over the first 48h, while refrigerated samples provided consistent plasma glucose results over the same time period (decrease of 0.26±0.23 mmol/L; 6±5%). The results from this study provide new insights into optimal blood sample handling and processing for glucose analysis in sea turtles, show the suitability of the POC glucometer as a rapid diagnostic test, and confirm the reliability of plasma glucose measurements using refrigeration. These findings emphasize the need to consider pre-/analytical factors when interpreting blood glucose results from loggerhead sea turtles.

## Introduction

The loggerhead sea turtle (*Caretta caretta*) is a worldwide distributed marine turtle species, currently classified as “vulnerable” by the International Union for Conservation of Nature, with an overall decreasing population trend [1]. The Northwest Atlantic population, from which this study population originates, is listed as threatened by the United States Endangered Species Act [2]. A large number of stranded loggerhead sea turtles are treated and monitored each year by rehabilitation facilities around the world [3]. Laboratory diagnostics, such as blood glucose measurements, are routinely used for diagnosis, prognosis, and patient monitoring. The monitoring of blood glucose concentrations in human and small animal medicine represents a cornerstone for critically-ill patient care, similar to its diagnostic utility in reptile medicine.

Glucose is the most abundant monosaccharide in vertebrates and is a vital metabolic fuel that provides tissues with energy when oxidized [4]. Blood glucose concentrations are regulated by a delicate balance of mechanisms involving hormones, glucose uptake, and oxidation [5,6]. In reptiles, blood glucose variations can occur due to alterations in metabolic rate, physiological changes, and environmental factors [7]. Physiological changes in blood glucose have been described in many reptile species, including changes associated with body size, reproductive status, temperature, sex, and season [7]. Reptile blood glucose measurements generally vary between 1.4 and 8.3 mmol/L and tend to be lower than blood glucose concentrations of birds and mammals [7,8].

Blood glucose concentration can be measured by clinical chemistry analyzers and point-of-care (POC) devices. POC glucometers are cost-effective, easy to use, require a small sample volume, and provide near real-time results. Conversely, clinical chemistry analyzers are more expensive and have a longer turnaround time, but are considered the gold standard methodology [9,10]. POC devices are popular in human and veterinary medicine due to the aforementioned reasons, but care should be taken when used in non-domestic species due to physiological and biochemical differences across taxa that can influence results, and the need to establish methodology-specific reference intervals. Validation of the precision of each POC device should always be performed [11].

Blood glucose can be measured in whole blood, serum, or plasma; however, these results are not interchangeable by sample type and/or species [9,10]. Glucose concentrations in serum/plasma and whole blood differ because the glucose content is measured in the aqueous component of the sample. Since the amount of water in mammalian red blood cells (RBC) (77%) is less than that in plasma (93%), the amount of glucose in whole blood is less than within an equivalent volume of serum or plasma [9]. To standardize and simplify the comparison of blood glucose results between methods, the International Federation of Clinical Chemistry and Laboratory Medicine recommends glucose concentrations to be reported as plasma equivalent values [12]. Therefore, POC glucometers that measure glucose in whole blood convert the result to a plasma equivalent value by using a specific built-in equation [9].

Although often underestimated in its importance, the pre-analytical phase is a critical component of the laboratory analysis and accounts for approximately 46–68% of laboratory errors [10]. Studies on the pre-analytical phase of sea turtles are limited [13]. Pre-analytical variables include physiological factors, aspects of specimen sampling and processing, and sample interferences. These include intrinsic (e.g., species, age, sex, nutritional and reproductive status) and extrinsic factors (e.g., season, husbandry, diet) [10].

Various aspects of specimen sampling and processing relate to changes in blood glucose concentrations that result from sample type, methodology, and processing techniques. Prandial status, partial pressure of oxygen (pO_2_), and tissue perfusion can influence blood glucose concentrations at different sampling sites [9,10]. Furthermore, sampling site can also influence biochemical analyses in chelonians due to dilution of blood with lymph, particularly with samples collected from the coccygeal and subcarapacial sinus; hence the external jugular vein is the preferred peripheral vessel for blood collection in these animals [14].

Time of sample processing after collection is a critical aspect of the pre-analytical phase, as *in vitro* blood glucose concentrations are unstable due to the ongoing glycolysis by blood cells when in continued contact with plasma, resulting in a steady decrease of glucose concentrations of approximately 5–7% per hour at room temperature in humans [15]. Interferences are variables that at specific concentrations can affect the methodology of glucose analysis, such as packed cell volume (PCV), lipemia, blood pH, hemolysis, pO_2_, and certain drugs in humans and mammals [9,10]. In mammals, PCV reportedly can influence glucose measurements of various POC glucometers [16–21].

The objective of this study was to investigate pre-analytical factors on whole blood and plasma glucose measurements in loggerhead sea turtles by evaluating the effects of storage (refrigeration) up to 48h after sampling and of PCV on whole blood glucose analysis by POC glucometer (time series n = 13); and by evaluating the effects of storage (room temperature and refrigeration) on plasma glucose concentrations using a dry slide chemistry analyzer (DCA) at various conditions: immediate processing and delayed plasma separation from erythrocytes at 24h and 48h (time series n = 14). The results from this study serve to establish recommendations for optimal blood sample handling and processing for glucose analysis in loggerhead sea turtles with the goal to obtain the most accurate results for data interpretation.

## Materials and methods

### Ethics statement

This study was carried out in accordance with all state, federal, international, and institutional guidelines. Data were collected under Florida Fish and Wildlife Conservation Commission (FWC) Marine Turtle Permits 021, 086, and 205, following FWC animal welfare guidelines, and UF IACUC# 201706823. All sampling procedures were reviewed by agency personnel prior to obtaining these permits. Trained staff of Loggerhead Marinelife Center (LMC) were responsible for sample collection of the sea turtles included in this study.

### Animals and study design

Blood was collected from nine nesting female loggerhead turtles on Juno Beach, Florida (from 26.920405°N, –80.065544°W to 26.836529°N, –80.0041335°W) during their nesting fixed action pattern. Additionally, blood samples from immature (n = 2) and adult (n = 3) loggerhead sea turtles undergoing rehabilitation at Loggerhead Marinelife Center (26.884511°N, –80.056144°W; Juno Beach, Florida, USA) were collected during hospitalization. All study animals were active, alert, behaved clinically normally as considered for nesting or rehabilitating turtles, respectively, and physical examination did not raise any concerns for any active underlying conditions. All rehabilitated turtles were successfully released. Data on sample dates, morphometrics, and clinical information of study turtles is included in S1 Table. All animals were manually restrained, and the venipuncture site (external jugular vein) was disinfected with alternating applications of betadine (Betadine® Solution Swabsticks, Purdue Products L.P., Stamford, Connecticut, USA) and 70% isopropyl alcohol followed by blood collection using 10 mL syringes and 1.5” 21-gauge needles. Approximately 1.5–2 mL of whole blood was placed in each of six lithium heparin 3 mL Vacutainer® tubes (Becton, Dickinson and Company, Franklin Lakes, New Jersey, USA) per turtle and gently inverted ten times to mix.

Blood from one lithium heparin Vacutainer® tube was kept refrigerated (1–2°C) for the entire time series, and blood glucose concentrations were determined at 0h (baseline), 2h, 4h, 6h, 8h, 10h, 12h, 14h, 16h, 18h, 20h, 22h, 24h, 36h, and 48h using the EasyTouch® glucose monitoring system (MHC® Medical Products, Fairfield, Ohio, USA). Samples from 13 turtles were used in this time series analysis. Whole blood was well mixed (carefully inverted ten times) before each glucometer measurement, and test strips were adequately filled per the manufacturer’s recommendations. EasyTouch® low and high control solutions were used as quality control (QC) before running each glucometer time series.

Whole blood from the other five lithium heparin Vacutainer® tubes was treated as follows: one sample (baseline) was processed as soon as possible (i.e., within fifteen minutes of collection); two samples were kept refrigerated (1–2°C) for 24h and 48h, respectively, and then processed; and two samples were kept at room temperature (22–24°C) for 24h and 48h, respectively, and then processed. All five whole blood samples were centrifuged for eight minutes at 4,200 G (5,000 rpms) using the LW Scientific C5 centrifuge (LW Scientific, Lawrenceville, Georgia, USA), the plasma was separated into cryovials, and immediately frozen at -80°C. All frozen plasma samples were analyzed for plasma glucose immediately after thawing using the IDEXX Catalyst Dx® (IDEXX® Laboratories, Inc., Westbrook, Maine, USA), a DCA, within five days of collection. Samples from all 14 study turtles were used in this time series analysis.

Packed cell volume was determined at time of blood processing from all 14 turtles at baseline (time 0h) using well-mixed heparinized whole blood. After gentle mixing, whole blood was added to a capillary tube; one end was sealed with a small amount of clay material. The capillary tube was centrifuged for eight minutes at 4,200 G using the LW Scientific C5 centrifuge (LW Scientific, Lawrenceville, Georgia, USA) with microcapillary tube inserts. Following centrifugation, the PCV was determined using a microhematocrit reader card. Hemolysis, lipemia, and dilution of blood with lymph were not apparent in any of the samples.

### Statistical analysis

All data were analyzed using R 3.6.0 [22] with the R packages epiR [23], lme4 [24], lmerTest [25], and lattice [26]. Agreement between results from the POC glucometer and the DCA were assessed using the Bland-Altman plot [27] and Lin’s concordance correlation coefficient (CCC) [28,29], with DCA results being treated as the gold standard methodology. CCC evaluates accuracy (C_b_, bias correction factor) and precision (r, Pearson’s correlation) of the agreement between methods [28]. The limits of agreement (LOA) were determined from the Bland-Altman plot by ±1.96 standard deviation of the mean difference between methods.

Linear mixed-effects models (LMMs) were used to assess the influence of time, PCV, and storage method on consecutive glucose measurements. To account for non-independence of within-subject variation, “the individual turtle” was considered a random effect in all analyses. Linear models (LMs) were used to assess the effect of PCV at baseline and the interaction between PCV and physiological condition (nesting or rehabilitating).

To evaluate the effect of time on the POC glucometer and considering the exponential decrease of glucose, “time” and “time squared” were used as fixed effects in the LMM. To evaluate the influence of PCV on the POC glucometer measurements, an LMM model using “time,” “time squared” and “PCV” as fixed effects was constructed. Additionally, to further understand the effect of PCV, LMs were used to assess the influence of PCV on glucose measurements at baseline, and to explore the interaction between PCV values and physiological condition. Finally, to evaluate the influence of delayed processing and storage method on DCA results, “time” and “storage method” were the fixed explanatory variables of the LMM. Analyses were performed using either the measured concentration (mmol/L) or a percentage from the baseline (0h).

The presented p-values are based on likelihood ratio tests. For all samples with concentrations below the limit of quantification (1.10 mmol/L for the EasyTouch® glucometer and 0.55 mmol/L for the IDEXX Catalyst Dx®), 1.05 mmol/L and 0.50 mmol/L were used in the calculations for the POC glucometer and DCA, respectively. All results were considered significant if p<0.05. For descriptive statistics, mean ± standard deviation (SD) are reported for normally distributed values, and the median, 25%, and 75% quartiles are reported for non-normally distributed data. Estimated coefficient or difference ± standard error (SE) are reported for the fixed effects in LMMs and LMs.

## Results

### Point-of-care glucometer validation

POC glucometer QC results averaged (mean ± SD) 3.10±0.26 mmol/L (range: 2.33–3.55 mmol/L) for the low control, and 18.07±1.38 mmol/L (range: 14.87–20.26 mmol/L) for the high control. The acceptable range for the low control solution was 1.67–3.55 mmol/L; and for the high control solution was 14.49–21.09 mmol/L. All QC solutions fell within the acceptable range, indicating accurate readings.

**Fig 1:**
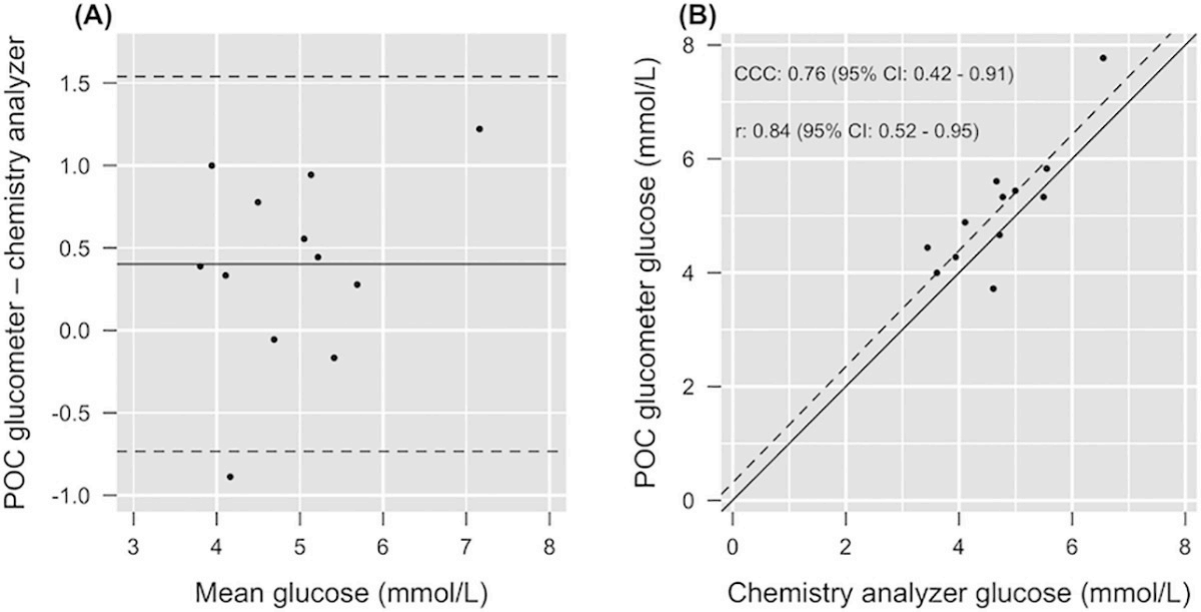
Validation of a point-of-care glucometer using whole blood against the gold standard of a dry slide chemistry analyzer using plasma from loggerhead sea turtles (*Caretta caretta*). (A) Bland-Altman plot of the difference between methods; solid line shows the mean difference (+0.4 mmol/L) and dashed lines identify the limits of agreement (-0.73 and +1.54 mmol/L). (B) Illustration of the concordance correlation; the identity line (solid, y = x) and the linear regression line (dashed) are shown. The graph illustrates strong accuracy (C_b_ = 0.90) and good precision (r = 0.84), with systematic bias. The concordance correlation coefficient (CCC = 0.76) shows substantial agreement between methods.

Mean (± SD) glucose concentration of the POC glucometer using refrigerated whole blood and DCA using refrigerated plasma at baseline were 5.22 ± 1.04 mmol/L (range: 3.72–7.77 mmol/L) and 4.70 ± 0.88 mmol/L (range: 3.44–6.55 mmol/L), respectively. The Bland-Altman plot indicated a small systematic positive bias, with the EasyTouch® glucometer giving slightly higher data than the DCA (mean of difference: +0.4 mmol/L, LOA: -0.73 and +1.54 mmol/L) (Fig 1A). Lin’s concordance (CCC = 0.76) showed substantial agreement between methods, with a very strong linear association (r = 0.84), and good accuracy (C_b_ = 0.90) (Fig 1B).

### Time series effects on point-of-care whole blood glucose measurements

**Fig 2:**
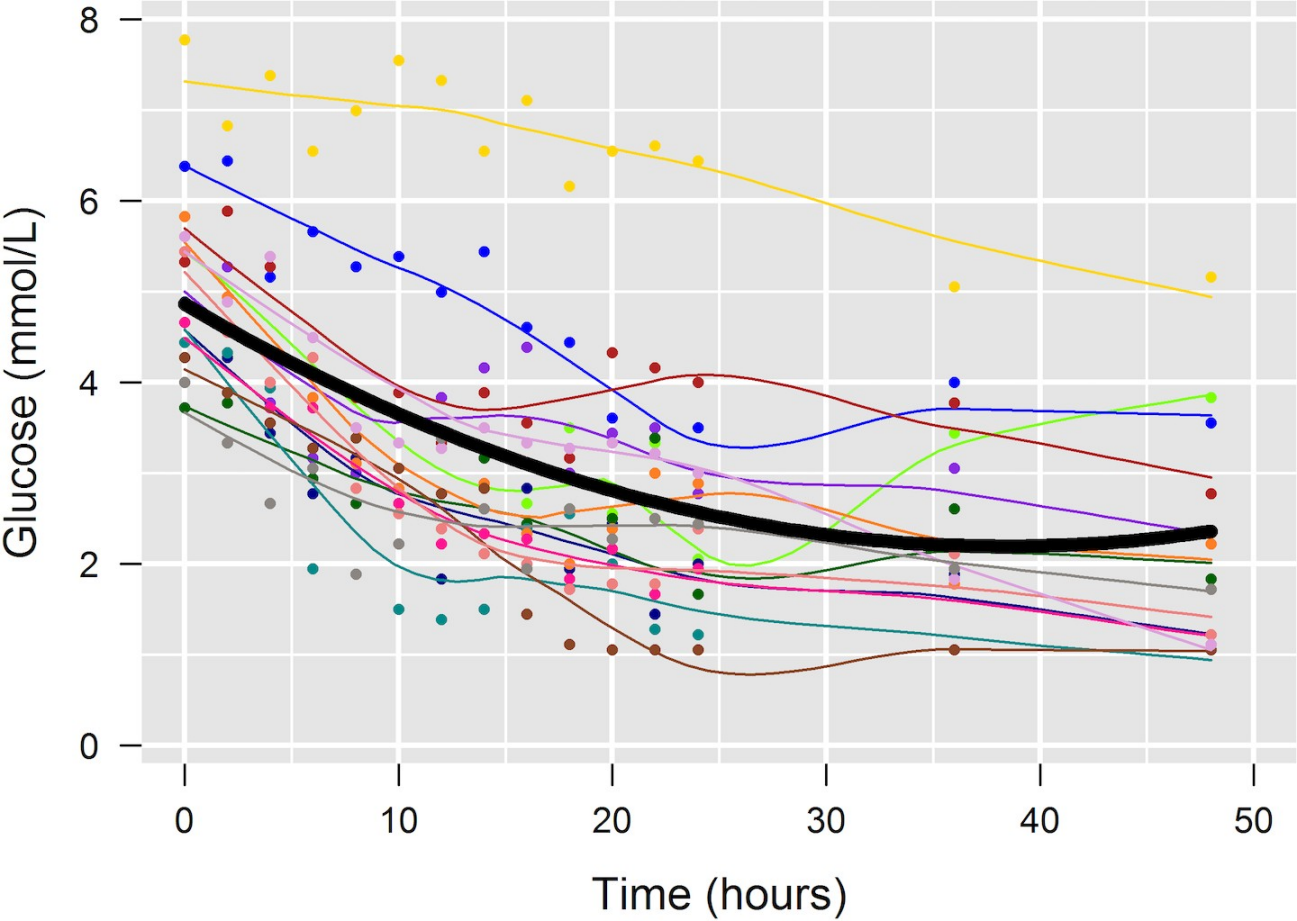
Effects of time on blood glucose measured by a point-of-care glucometer using refrigerated whole blood from loggerhead sea turtles (*Caretta caretta*). Different colors identify time series of individual turtles; dots represent the actual measured concentrations, and the colored lines represent the fitted LOESS smooth curves. The thick dark line represents the decrease in glucose as estimated by the linear mixed effect model, based on the formula: -0.139×T+0.001809×T^2^; where T is time in hours.

For all models in this study, plots of residuals against fitted values revealed no apparent deviations from normality or homogeneity of variance. The influence of time on the refrigerated whole blood POC glucometer measurements was significant (p<0.0001) (Table 1). The results showed an exponential decrease, with a faster decrease within the first 10h after sampling (Fig 2). The measured decrease in glucose is given in Table 2. Glucose decreased on average (mean ± SD) by 0.41±0.47 mmol/L (8±9%) within the first 2h; 1.95±0.96 mmol/L (39±19%) within the first 12h; 2.55±0.71 mmol/L (51±16%) within the first 24h; and 3.08±0.84 mmol/L (60±17%) within the first 48h.

**Table 1:** Fixed effects result for the point-of-care glucometer over time using refrigerated whole blood from loggerhead sea turtles (*Caretta caretta*).

Fixed effects	C [mmol/L]	SE [mmol/L]	C [%]	SE [%]	p-value
Time	-0.139	0.009	-2.755	0.201	<0.0001
Time squared	0.002	0.000	0.036	0.004	<0.0001

Fixed effects coefficient (C), standard error (SE), and p-value of the linear mixed-effect models for whole blood glucose measurements using the point-of-care glucometer.

**Table 2:** Descriptive statistics of blood glucose concentrations measured by a point-of-care glucometer using refrigerated whole blood from loggerhead sea turtles (*Caretta caretta*).

Time points (h)	Measured glucose (mmol/L)	Total difference (mmol/L)	Total difference (%)	Time point difference (mmol/L)	Time point difference (%)
0	5.22±1.04				
2	4.79±1.07	-0.41±0.47	-8±9	-0.41±0.47	-8±9
4	4.39±1.31	-0.89±0.54	-18±11	-0.48±0.63	-10±11
6	3.84±1.24	-1.36±0.60	-27±13	-0.46±0.74	-9±15
8	3.41±1.37	-1.79±0.68	-36±14	-0.43±0.59	-9±12
10	3.33±1.57	-1.88±0.85	-38±17	-0.09±0.31	-2±6
12	3.25±1.53	-1.95±0.96	-39±19	-0.07±0.52	-1±11
14	3.37±1.38	-1.84±0.86	-36±17	0.12±0.50	2±10
16	3.09±1.47	-2.13±0.84	-42±17	-0.24±0.61	-6±13
18	2.79±1.31	-2.43±0.75	-48±16	-0.31±0.60	-6±12
20	2.89±1.34	-2.33±0.84	-46±17	0.10±0.60	2±12
22	2.88±1.46	-2.34±1.01	-46±22	-0.01±0.53	0±12
24	2.67±1.36	-2.55±0.71	-51±16	-0.21±0.64	-5±15
36	2.54±1.17	-2.68±0.84	-52±17	-0.13±0.78	-2±14
48	2.14±1.28	-3.08±0.84	-61±17	-0.40±0.51	-8±10

All results are given in mean ± standard deviation. The total difference corresponds to the change when compared to time zero; time point difference corresponds to the change with the previously measured time point.

### Effects of packed cell volume on point-of-care whole blood glucose measurements

Packed cell volume of all study turtles ranged from 0.06 to 0.29L/L, with a median of 0.24L/L (25% and 75% quartile: 0.22 and 0.25L/L). The individual’s physiological condition influenced PCV data significantly (p = 0.004), with nesting turtles having significantly higher PCV (coefficient ± SE: +0.07±0.02L/L) than rehabilitating turtles. The PCV of rehabilitating turtles ranged from 0.06 to 0.24L/L (median: 0.22L/L; 25% and 75% quartile: 0.13 and 0.24L/L), and nesting turtles’ PCV ranged from 0.21 to 0.29L/L (mean ± SD: 0.25±0.031L/L). While physiological condition influenced PCV, there was no significant effect of condition on glucose at time zero (p = 0.398).

When all animals for this analysis were included (n = 13), PCV showed a significant influence (p = 0.033) on the decrease of glucose over time (coefficient ± SE: -0.12±0.05 mmol/L) when using the POC glucometer with whole blood. However, if only animals with PCV>0.1L/L were included (n = 12), PCV did not affect whole blood glucose (p = 0.845). Similarly, at baseline, PCV had an inversely proportional significant influence on glucose measurements (p = 0.010), as PCV increased, glucose decreased (coefficient ± SE: -0.11±0.04 mmol/L). This effect was only significant if the outlier (PCV = 0.06 L/L) was included in the model. Once the outlier was removed from the model, the effect of PCV on glucose at baseline was not significant (p = 0.462).

### Effects of storage method and delayed processing on dry slide chemistry plasma glucose measurements

**Fig 3:**
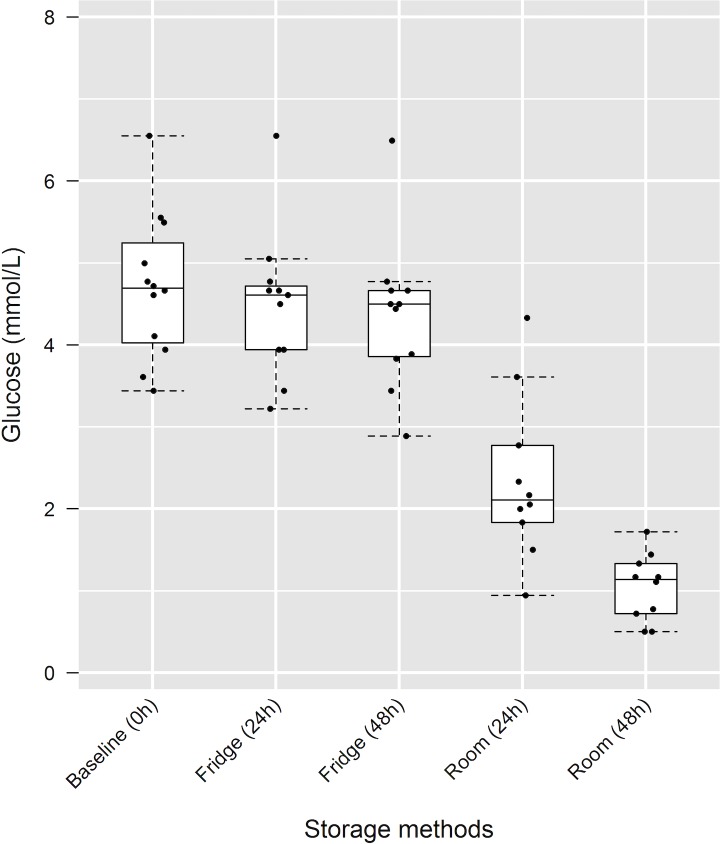
Effects of delayed processing and storage methods on plasma glucose measurements using the dry slide chemistry analyzer in loggerhead sea turtles (*Caretta caretta*). Box and whisker plots of the glucose measurements at different processing times (0h, 24h, and 48h) and with different storage methods of whole blood before analysis (refrigerated [fridge] or room temperature [room]). Each dot represents an individual turtle; the boxes show the respective 25^th^ and 75^th^ percentiles, the line within the box represents the median values, and the whiskers the lowest and highest data points still within 1.5 times the interquartile range of the respective lower and upper quartiles.

Storage method (refrigerated vs. room temperature) had a significant effect on plasma glucose measurements using the DCA (Fig 3), with samples kept at room temperature resulting in significantly lower glucose data (p<0.0001) (Table 3). As shown in Table 3, samples kept refrigerated did not present a significant effect due to delayed sample processing. The samples kept at room temperature, however, decreased on average (mean ± SD) 2.26±0.90 mmol/L (49±17%) over the first 24h, and 3.57±0.89 mmol/L (77±9%) over the first 48h.

**Table 3:** Plasma glucose measurements and generalized linear mixed-effect model estimates for the dry slide chemistry analyzer at refrigerated (fridge) and room (room) temperatures in loggerhead sea turtles (*Caretta caretta*).

Storage	TP (h)	Measured glucose (mmol/L)	Total difference (mmol/L)	Total difference (%)	Estimated difference (mmol/L)	p-value
-	0	4.70±0.88	-	-	-	-
Fridge	24	4.49±0.90	-0.15±0.16	-3±3	-0.17±0.21	0.3245
Fridge	48	4.37±0.92	-0.26±0.23	-6±5	-0.29±0.21	0.0775
Room	24	2.35±0.99	-2.26±0.90	-49±17	-2.30±0.22	<0.0001
Room	48	1.04±0.41	-3.57±0.89	-77±9	-3.61±0.22	<0.0001

Time points (TP) in hours (h). All results are given in mean ± standard deviation, except estimated difference, which is given in mean ± standard error. The total difference corresponds to the change when compared to baseline. The estimated difference corresponds to the results of the generalized linear mixed-effect model.

## Discussion

This study demonstrates the importance of considering pre-analytical and analytical factors when interpreting glucose data from loggerhead sea turtles, as differences in analytical methodology and storage conditions can result in variability of results or spurious decreases. The effects of pre-analytical factors can potentially alter clinical decisions in rehabilitating patients or interpretation of population-level data. Blood glucose concentrations in loggerhead sea turtles vary greatly depending on age and health status, with adults, particularly adult nesting females, typically having lower glucose compared to younger life stage classes [30–35], and foraging loggerheads having higher glucose than nesting or stranded turtles [36,37]. The comparison of blood glucose data across studies should be made with caution. The time between sampling and processing varies considerably across studies, as does refrigeration of sample between collection and analysis, the origin of study animals (captive or wild), and analytical methodology. Studies of the pre-analytical phase in sea turtles are limited [13]; however, this phase is known to result in interferences with glucose measurements and should be considered when interpreting results and comparing data with the literature.

Hypoglycemia has been reported in stranded and chronically debilitated turtles and, in addition to other biochemical analytes, plasma glucose equal to or higher than 3.05 mmol/L upon admission was predictive of survival [36]. Therefore, the accuracy of glucose measurements in admitted turtles with chronic debilitation or other stranding causes is crucial for triaging, medical management, and monitoring during rehabilitation. The glucose measurements at baseline (5.22±1.04 mmol/L and 4.70±0.88 mmol/L for the POC glucometer and DCA, respectively) were comparable to previously published data for nesting and rehabilitating turtles [36,37], with no significant difference in rehabilitating versus nesting turtles.

### Point-of-care glucometer validation

Point-of-care glucometers are portable, provide fast and cost-effective results, are easy to handle, and offer a new diagnostic procedure during field assessments and surveys of sea turtles [35]. However, proper assay validation of a new diagnostic method is necessary for appropriate result interpretation. The EasyTouch® glucose monitoring system used in this study had previously been validated in green turtles (*Chelonia mydas*) [38]. Although blood glucose in green turtles may be different from loggerhead sea turtles during health and disease, there was very strong correlation between methods (POC glucometer vs. DCA) in green turtles [38]. EasyTouch® glucose analysis showed a systematic positive bias in loggerhead sea turtles in this study, overestimating glucose measurements when compared to the DCA. These findings are similar to green turtles [38] and indicate that results from the POC glucometer were acceptable overall, but the systematic overestimation of glucose should be considered when making clinical decisions or comparisons to other published glucose data in loggerhead sea turtles. For example, hypoglycemia may not be detected, or a patient diagnosed with hypoglycemia by glucometer is likely much more hypoglycemic when comparing plasma glucose. These considerations should be kept in mind when evaluating POC glucometer data from individual patients.

### Time series effects on point-of-care whole blood glucose measurements

POC glucose measurements of refrigerated whole blood were significantly influenced by time, with glucose decreasing substantially over 48h. Compared to decreases of DCA plasma glucose stored refrigerated over time, this decrease was much more pronounced using the glucometer. Considerations for the more pronounced decrease in glucose measurements using the POC glucometer include differences in sample handling (e.g., thorough mixing of the blood sample for glucometer samples prior to each reading could have increased contact of erythrocytes with plasma glucose and resulted in increased consumption) or in analytical methodology (e.g., POC glucometer vs. DCA). Similar to DCA samples, separate vials may have been useful for different time point measurements of the POC glucometer as well, to account for the impact of handling. Different tubes for each time point would have allowed to more accurate evaluation of the effects of time on the POC glucometer results. Furthermore, strictly controlled environmental temperatures could also have improved the comparability of results, although the temperature variability was minimal in the air-conditioned in-house laboratory.

The decrease noted within the first 2h for the POC glucometer (0.41±0.47 mmol/L; 8 ± 9%) may be clinically relevant and was within the total allowable error of 10% for glucose below the reference range based on the American Society for Veterinary Clinical Pathology (ASVCP) guidelines [39]. Hence, refrigerated whole blood samples stored for 2h or longer did not produce reliable blood glucose results by EasyTouch®. Given that the POC glucometer tends to overestimate plasma glucose [38], glucose measurements close to the lower end of published reference intervals for loggerhead sea turtles [31–35,37] should be considered hypoglycemic or at risk for hypoglycemia. These considerations emphasize the need for POC glucometer samples to be processed immediately after sample collection.

### Packed cell volume’s influence on point-of-care whole blood glucose measurements

Most (85%; n = 11 of 13 turtles) of the PCV data in this study were within 0.20L/L and 0.29L/L and in agreement with previously published data for nesting and rehabilitating turtles [37,40]. Two turtles were considered anemic, with PCV data 0.06L/L and 0.13L/L, respectively. Packed cell volume significantly influenced glucose measurements over time when all animals were included, but when the most extreme outlier (PCV = 0.06L/L) was removed from the model, PCV did not affect glucose. Therefore, the data included in this study are considered insufficient to fully assess the effect of PCV on glucose measurements. A larger dataset with a broader range of PCV data would be necessary to assess the relationship between PCV at baseline and glucose using a POC glucometer.

Although the progressive decrease of glucose in uncentrifuged whole blood samples without glycolysis inhibitors has been attributed to blood cell consumption, in particular by RBC in other species [41], the influence of PCV on consecutive glucose measurements has not been verified in some studies that analyzed delayed processing in serum and plasma samples [42,43]. In human uncentrifuged lithium-heparinized plasma and serum samples, the decrease of glucose over time was dependent on the baseline number of RBC, white blood cells (WBC), neutrophils and monocytes, but not baseline PCV [43]. In contrast, variable PCV data have been shown to influence the results of POC glucometers in humans [16] and several animal species [17–21]. In humans, the most pronounced effects were noted at low and high PCV data, which generally resulted in higher or lower glucose measurements, respectively [9,16]; similar effects have been described in domestic species [17–19]. On the other hand, some studies in cats (*Felis catus*) and horses (*Equus caballus*) have found no association between PCV data and POC glucometer glucose concentrations [44,45]. According to ASVCP guidelines, veterinarians should interpret glucose concentrations from a POC glucometer with caution in anemic and hemoconcentrated patients [9]. In addition to PCV data, inclusion of RBC and WBC counts in the analyses could contribute to further understanding of the influence of glucose consumption by different blood cells over time in future studies. The narrow range of PCV data in this study, with the majority of turtles presenting normal PCVs, might explain the difficulty to discern any relationship between PCV and glucose measurements accurately. Furthermore, loggerhead PCV data are considered low when compared to those from clinically normal mammalian species. The accuracy of the EasyTouch® glucometer in humans showed unacceptable results when PCV was below 0.30L/L, with approximately 26% of the POC glucometer readings >20% of the reference values [46]. Additionally, Tang et al. [16] reported a consistent overestimation of glucose with a variety of POC glucometers at low PCV (0.19L/L) in humans. All of the samples used in this study had PCVs below 0.30L/L and would be considered anemic in the human trials. Nevertheless, the results between DCA and EasyTouch® showed a very strong correlation, although the POC glucometer presented a systematic positive bias. It is unclear if the consistent overestimation seen by the POC glucometer is due to the instrument or the “low” PCV values of our samples. Given these considerations and the low sample size for this aspect of our study, further studies are needed to understand the influence of PCV/RBC on *in vitro* glycolysis and on POC glucometer measurements, particularly in non-mammalian species with nucleated erythrocytes, as energy metabolism varies between mammalian erythrocytes and nucleated RBC [47].

### Effects of storage method and delayed processing on dry slide chemistry plasma glucose measurements

Storage method had significant effects on DCA plasma concentrations over time, with samples kept at room temperature presenting significantly lower results than samples that were stored by refrigeration. Accelerated *in vitro* glycolysis at room temperature is likely attributed to the fact that glucose consumption is a substrate- and temperature-dependent enzymatic reaction, which may be variably affected by species. This influence of *in vitro* glycolysis is well documented in mammals [15]; however, the extent of *in vitro* glucose consumption has not been widely investigated in reptiles. Previous studies in reptiles evaluated delayed plasma separation in samples under refrigeration [13,48]; to the authors’ knowledge, this is the first study investigating the effects of *in vitro* glycolysis of samples stored at room temperature in sea turtles.

Very few studies exist that evaluated plasma glucose concentrations at room temperature and for at least 24h in various species. In humans, significant plasma glucose consumption was present when whole blood samples were kept at room temperature and uncentrifuged, with reported decreases of 72% [15] and 3.98 mmol/L [49] within 24h. In contrast, whole blood samples of psittacine birds kept under similar conditions and time decreased only by 2.40 mmol/L or 16% [50]. In the present study, glucose decreased 2.26±0.90 mmol/L (49±17%) and 3.57±0.89 mmol/L (77±9%) when whole blood was stored at room temperature for 24h and 48h, respectively, showing a faster decrease in plasma glucose at 24h when stored at room temperature than compared to psittacine birds [50], but lower than in humans [15,49]. Despite this lower glucose decrease in sea turtles compared to humans, possibly from the lower metabolic rate of reptiles [51], it was significant over 24h; consequently, plasma glucose data from whole blood samples stored for 24h at room temperature and then being centrifuged for plasma harvest should not be used for glucose measurements.

While the decrease in plasma glucose concentrations at room temperature over time presented statistical and presumptive clinical significance, whole blood samples kept under refrigeration and then centrifuged for plasma harvest showed minimal non-significant decreases in glucose measurements. After 24h, glucose of loggerhead turtles in this study decreased by 0.15±0.16 mmol/L (3±3%), and after 48h by 0.26±0.23 mmol/L (6±5%). Previous studies in reptiles that evaluated plasma glucose over time with uncentrifuged, refrigerated whole blood samples reported a decrease of 10–60% in Burmese python (*Python bivittatus*) samples over 24h (60% decline in 4 out of 10 samples) [48], and 2% in 24h and 3% in 48h for loggerhead sea turtle samples [13]. All studies had significantly different initial glucose measurements (mean ± SD): 4.70±0.88 mmol/L (present study), 1.94±0.55 mmol/L (pythons) [48], and 9.32±1.00 mmol/L (loggerheads) [13]. The influence of the initial glucose concentration or other factors at the time of initial processing on the glucose consumption rate is undetermined to date. The differences found by Eisenhawer et al. [13] in plasma glucose from uncentrifuged, refrigerated whole blood samples at 24h and 48h were comparable to our results, especially when the total difference in mmol/L was considered rather than the percentage change. Physiological differences (e.g., life stage, nesting vs. rehabilitating turtles) could interfere with intra-erythrocytic glucose metabolism and *in vitro* glycolysis rate [13]. Furthermore, erythrocyte glucose consumption varies across species [52,53], as it depends on erythrocyte glucose permeability, as well as the metabolic pathways used for energy production by RBC [47,53]. These considerations could account for differences in loggerhead sea turtle *in vitro* glucose consumption when compared to other species.

The glucose decline with samples under refrigeration was not statistically or clinically significant. The decrease seen after 48h was still within the total allowable error for glucose below the reference range based on ASVCP guidelines [39]. Therefore, immediate refrigeration of whole blood samples of loggerhead sea turtles is highly recommended to ensure accurate results. Although the decrease in glucose was not statistically significant in our study, glucose will decline over time, even under refrigeration [54]. Immediate processing is necessary, especially in critically ill patients, or patients in which accurate glucose measurements are necessary for other reasons.

In human medicine, even a 30 minute storage period in an ice/water slurry is considered too long, as there could be substantial inter-individual differences in the *in vitro* glycolysis rate [54]. This needs to be considered by researchers working under field conditions (e.g., when sampling nesting turtles) in that proper accommodations should be made for immediate blood sample cooling and processing at the earliest possibility.

The gold standard samples (DCA at baseline) were stored frozen at –80°C; however, due to prolonged stability of glucose in frozen samples [55–57], the effect of freezing was not considered a concern in this study.

## Conclusions

The POC glucometer presented a systematic positive bias, overestimating glucose measurements when compared to the DCA. This systematic overestimation of glucose should be considered when making clinical decisions for individual patients but also when interpreting data from a group or population of loggerhead sea turtles. Refrigerated POC glucometer data significantly decreased over time, and the decline noted within the first 2h is considered clinically relevant, highlighting the need for immediate blood sample processing when using this analytical methodology. Storage method had significant effects on plasma glucose measurements, showing that whole blood samples destined for glucose analysis that are stored at room temperature or lost in transit should not be used for plasma glucose evaluation at any time point. Refrigerated whole blood samples provided reliable DCA plasma glucose results even after 48h of storage. Whole blood samples should be refrigerated immediately after collection and processed as soon as possible, considering that there is an ongoing decrease during storage even with refrigeration. The ideal sample for glucose analysis is separated as soon as possible from RBC after sample collection, transferred into a vial suitable for holding plasma, and either analyzed immediately in-house or shipped on cold packs when submitted to a diagnostic laboratory. The information provided herein emphasizes the need to consider pre-analytical and analytical factors when interpreting blood glucose data from loggerhead sea turtles in various clinical and research settings.

## Supporting information

S1 Table Sample date, size (minimum curved carapace length, CCLmin), life-stage class/sex, and condition of loggerhead sea turtles (Caretta caretta) included in this study. (DOCX)
