# Supplementary material for: Pre-/analytical factors affecting whole blood and plasma glucose concentrations in loggerhead sea turtles (Caretta caretta)
Source: PLoS One. 2020 Mar 3;15(3):e0229800. doi: 10.1371/journal.pone.0229800 (PMC7053744; doi:10.1371/journal.pone.0229800)
Supplement: S1 Table — (DOCX) [file pone.0229800.s001.docx]

| **S1 Table**. Sample date, size (minimum curved carapace length, CCL_min_), life-stage class/sex, and condition of loggerhead sea turtles (*Caretta caretta*) included in this study. | | | | | |
| --- | --- | --- | --- | --- | --- |
| ID | Sample date | CCL_min_ (cm) | Life-stage class/sex | Condition | PCV |
| 876 | 22 Jun 2018 | 88.5 | Mature/female | Gravid | – |
| 877 | 25 Jun 2018 | 95.3 | Mature/female | Gravid | 25 |
| 891 | 29 Jun 2018 | 91.1 | Mature/female | Gravid | 23 |
| 898 | 6 Jul 2018 | 108.1 | Mature/female | Gravid | 22 |
| 908 | 9 Jul 2018 | 90.8 | Mature/female | Gravid | 29 |
| 929 | 13 Jul 2018 | 82.9 | Mature/female | Gravid | 25 |
| No ID | 16 Jul 2018 | – | Mature/female | Gravid | 21 |
| 941 | 18 Jul 2018 | 81.3 | Mature/female | Gravid | 26 |
| 944 | 19 Jul 2018 | 84.3 | Mature/female | Gravid | 29 |
| CC1810 | 24 Jul 2018 | 97.0 | Mature/male | Chronic debilitation | 24 |
| CC1815 | 30 Jul 2018 | 60.7 | Immature/unknown | Chronic debilitation | 24 |
| CC1770 | 30 Jul 2018 | 95.6 | Mature/female | Chronic debilitation | 22 |
| CC1822 | 24 Oct 2018 | 70.5 | Immature/unknown | Chronic debilitation | 13 |
| CC1904 | 19 Mar 2019 | 90.2 | Mature/male | Boat strike/anemia | 6 |
